# Artificial Intelligence-Based Classification of Multiple Gastrointestinal Diseases Using Endoscopy Videos for Clinical Diagnosis

**DOI:** 10.3390/jcm8070986

**Published:** 2019-07-07

**Authors:** Muhammad Owais, Muhammad Arsalan, Jiho Choi, Tahir Mahmood, Kang Ryoung Park

**Affiliations:** Division of Electronics and Electrical Engineering, Dongguk University, 30 Pildong-ro 1-gil, Jung-gu, Seoul 04620, Korea

**Keywords:** Artificial intelligence (AI), deep learning, endoscopic video analysis, residual network (ResNet) and long short-term memory (LSTM) model, classification of multiple gastrointestinal (GI) diseases

## Abstract

Various techniques using artificial intelligence (AI) have resulted in a significant contribution to field of medical image and video-based diagnoses, such as radiology, pathology, and endoscopy, including the classification of gastrointestinal (GI) diseases. Most previous studies on the classification of GI diseases use only spatial features, which demonstrate low performance in the classification of multiple GI diseases. Although there are a few previous studies using temporal features based on a three-dimensional convolutional neural network, only a specific part of the GI tract was involved with the limited number of classes. To overcome these problems, we propose a comprehensive AI-based framework for the classification of multiple GI diseases by using endoscopic videos, which can simultaneously extract both spatial and temporal features to achieve better classification performance. Two different residual networks and a long short-term memory model are integrated in a cascaded mode to extract spatial and temporal features, respectively. Experiments were conducted on a combined dataset consisting of one of the largest endoscopic videos with 52,471 frames. The results demonstrate the effectiveness of the proposed classification framework for multi-GI diseases. The experimental results of the proposed model (97.057% area under the curve) demonstrate superior performance over the state-of-the-art methods and indicate its potential for clinical applications.

## 1. Introduction

Different types of gastrointestinal (GI) diseases, such as colorectal cancer and tumor, are the leading cause of death in the USA [1]. According to the American Cancer Society, approximately 76,940 people lost their lives in 2016 owing to different types of cancers in the GI tract [1]. The effective diagnosis of such GI diseases is a tedious and time-consuming task. Most of the small GI lesions remain imperceptible during the early stages, which ultimately evolves into a fatal ailment. Therefore, it is essential to develop computerized approaches that can assist the physicians in effective diagnosis and treatment. Therefore, substantial efforts were focused over the past few decades to develop artificial intelligence (AI)-based computer-aided diagnosis (CAD) tools and applications in various medical fields [2,3,4]. These fields include the detection of brain tumor [5], classification of different types of skin cancers, diagnosis in radiation oncology, diabetic retinopathy, histologic classification of gastric biopsy, and endoscopy [6,7,8,9,10,11,12,13,14,15].

In the field of endoscopy, the recent AI-based CAD tools utilize the strength of deep learning (a set of advanced machine learning algorithms) for the analysis of various types of endoscopic scans. In general, deep learning algorithms are used to extract the optimal representations of training data. A training algorithm optimizes the learnable parameters of the deep learning model [16]. Based on the optimal features extracted from the training dataset, the CAD tool can analyze the newly acquired clinical images prospectively. Artificial neural networks (ANNs) are the key component of such deep learning-based image analysis tools that logically emulates the structure and activity of the brain neurons on a computer. Various types of ANNs were proposed, including convolutional neural networks (CNNs), in the field of image recognition [16,17]. However, all the supervised ANN-based image recognition methods require a training dataset, which is used to estimate the optimal network parameters for sufficient training. In the field of medical image analysis, similar training of an ANN model is performed, which is called supervised learning (SL). In SL, the available training dataset consists of both input images and appropriate output information. The primary portion of ANN model is the stack of multiple layers, which comprise of learnable filters with different values of size and depth. These layers extract the complex features from the available training dataset by using different learning algorithms. Finally, the ANN network learns from these features without using other handcrafted features [16,17]. After performing sufficient training, an ANN-based diagnostic framework demonstrates the best performance in various clinical applications.

This research primarily focuses on the analysis of different deep learning models used in the classification of GI diseases. We analyze in depth the performance of the most recent CNN models considering the following perspectives: (1) the importance of spatial and temporal features in the classification of GI diseases; (2) feature selection from different layers within a CNN network; (3) combining the CNN and long short-term memory (LSTM); and (4) analyzing the effects of the temporal features by considering different number of successive frames. Thus, we proposed a cascaded deep feature-based framework by combining the deep residual network (ResNet) and LSTM to obtain the best classification accuracy. Finally, we provide a novel spatiotemporal features-based pretrained model for the classification of multiple GI diseases, which is our primary contribution. We have also ensured that our pretrained model and the video indices of the experimental endoscopic videos are publicly available for other researchers [18].

The rest of this paper is organized as follows: The related studies on endoscopy for the detection and classification of different GI diseases are provided in Section 2, and a brief summary of our contribution to this research is explained in Section 3. In Section 4, a comprehensive description of the proposed classification framework for multiple GI diseases is presented. In Section 5, we illustrate the experimental setup and performance analysis of the proposed method to validate its performance and efficiency over the previous deep learning and handcrafted features-based methods. A discussion on certain important issues relevant to this paper is presented in Section 6. Finally, Section 7 draws a conclusion of our research work.

## 2. Related Works

In recent years, the strength of deep learning-based algorithms has been utilized in the field of endoscopy, including capsule endoscopy (CE), esophagogastroduodenoscopy (EGD), and colonoscopy [6,7,8,9,10,11,12,13,14,15]. To facilitate the physicians with the effective diagnosis of different GI lesions, several CNN-based CAD tools have been proposed in the literature. These CAD tools are capable of detecting and classifying even small lesions in the GI tract, which often remain imperceptible to the human visual system. Before the advent of deep learning methods, many previous studies have focused on the handcrafted feature-based methods, which mainly consider texture and color information.

Most of the previous studies have been carried out to perform the detection and classification of different type of GI polyps in the field of CE. Generally, these methods followed a common approach of the feature extracting and then classification to detect and classify the GI polyps. In [19], Karargyris et al. proposed a geometric and texture features based method for the detection of small bowel polyps and ulcers in CE. Log Gabor filters and the SUSAN edge detector was used to preprocess the images and, finally, the geometric features were extracted to detect the polyp and ulcer region. Li et al. [20] utilized the advantages of a discrete wavelet transform and uniform local binary pattern (LBP) with a support vector machine (SVM) to classify the normal and abnormal tissues. In this feature extraction approach, wavelet transform combines the capability of multiresolution analysis and uniform LBP to provide robustness to illumination changes, which results in better performance.

Similarly, another texture features-based automatic tumor recognition framework was proposed in [6] for wireless CE images. In this framework, a similar integrated approach was adopted based on LBP and discrete wavelet transform to extract the texture features of the scale and rotation invariants. Finally, the selected features were classified by using an SVM. Yuan et al. [21] proposed an integrated polyps detection algorithm by combing the Bag of Features (BoF) method with the saliency map. In the first step, the BoF method characterizes the local features by using a scale-invariant feature transform (SIFT) feature vectors with k-means clustering. Then saliency features were obtained by generating saliency map histogram. Finally, both BoF and saliency features were fed into the SVM to perform classification. Later, Yuan et al. [22] extended this approach with the addition of LBP, uniform LBP (ULBP), complete LBP (CLBP), and histogram of oriented gradients (HoG) features along with SIFT features for capturing more discriminative texture information. Finally, these features were classified by using SVM and Fisher’s linear discriminant analysis (FLDA) classifiers by considering different combinations of local features. The combination of SIFT and CLBP features with SVM classifier resulted in top classification accuracy.

Seguí et al. presented a deep CNN system for small intestine motility characterization [7]. This CNN-based method exploited the general representation of six different intestinal motility events by extracting deep features, which resulted in superior classification performance when compared to the other handcrafted features-based methods. Another CNN-based CAD tool was presented in [15] to quantitatively analyze the celiac disease in a fully automated approach by using CE videos. This proposed method utilized the advantages of a well-known CNN model (i.e., GoogLeNet) to distinguish between the normal and abnormal (i.e., diagnosed with celiac disease) patients. Thus, the effective characterization of the celiac disease resulted in better diagnosis and treatment when compared to the manual analysis of CE videos. In [12], a multistage deep CNN-based framework for hookworm (i.e., intestinal parasite) detection was proposed using CE images. Two different CNN networks, named as edge extraction network and hookworm classification network, were unified, which simultaneously characterized the visual and tubular patterns of hookworms.

In the field of EGD, a deep learning-based CAD tool was proposed for the diagnosis of *Helicobacter pylori* (*H. pylori*) infection [9]. In this proposed framework, two-stage CNN models were used. In the first stage, a 22-layers deep CNN was fine-tuned for the classification (i.e., positive or negative) of *H. pylori* infection. Then, in the second stage, another CNN was used to further classify the dataset (EGD images) according to eight different anatomical locations. Similarly, Takiyama et al. proposed another CNN-based classification model to categorize the anatomical location of the human GI tract [8]. This technique could categorize the EGD images into four major anatomical locations (i.e., larynx, esophagus, stomach, and duodenum) and three subcategories for the stomach images (upper, middle, and lower regions). A pretrained CNN architecture, named as GoogLeNet, was used for this classification problem, which demonstrated high classification performance. In a recent study by Hirasawa et al. [13], a fully automated diagnostic tool for gastric cancer was proposed by utilizing the detecting capability of deep CNN-based architectures. A single-shot multibox detector (SSD) architecture was used to detect early and advanced stages of gastric cancer from EGD images. The proposed method demonstrated substantial detection capability even for small lesions when compared to the conventional methods. The results of this study illustrated its practical usability in clinical practice for better diagnosis and treatment. However, it demonstrated certain limitations as only high-quality EGD images could be used from the same type of endoscope and endoscopic video system. 

Generally, the various deep learning-based methods demonstrate either the problem of over-fitting or under-fitting owing to the utilization of a large number of network parameters and the limited amount of data available in the training dataset. This problem degrades the system performance in a real-world scenario. A similar problem also occurs in the domain of medical image analysis owing to the unavailability of a sufficiently large training dataset. To address this issue, a transfer learning mechanism is often adopted in this domain. In the field of colonoscopy, Zhang et al. [10] used this approach for automatic detection and classification of colorectal polyps. A novel transfer learning approach was applied to train the two different CNN models for the source domain (i.e., nonmedical dataset) and then fine-tuning was performed for the target domain (i.e., medical dataset). Their method performed the polyp detection and classification tasks in two different stages. In the first stage, an image of interest (i.e., polyp image) was selected by using the CNN-based polyp detection model. In the second stage, another CNN model was further used to categorize the detected polyp image into either a hyperplastic polyp or an adenomatous colorectal polyp. The results of this study demonstrated that the CNN-based diagnoses achieved a higher accuracy and recall rate than endoscopist diagnoses. However, their method is not applicable for real-time colonoscopy image analysis owing to the use of multistage CNN models. Another study by Byrne et al. [14], presented a single deep CNN-based real-time colorectal polyp classification framework using the colonoscopy video images. In this study, a simple CNN model was trained to classify each input frame into one of four different categories, i.e., hyperplastic polyp, adenomatous polyp, no polyp, or unsuitable. The end-to-end processing time of this CNN model was 50 ms per frame, resulting in its applicability for the real-time classification of polyps. In another study [11], an offline and online three-dimensional (3D) deep CNN framework was proposed for automatic polyp detection. Two different 3D-CNNs, named as offline 3D-CNN and online 3D-CNN, were simultaneously used to exploit the more general representation of features for the task of effective polyp detection. In this complete framework, the offline 3D-CNN effectively reduced the number of false positives, whereas the online 3D-CNN was used to further improve the polyp detection. The experimental results showed that the 3D fully convolutional network was capable of learning more representative spatiotemporal features from colonoscopy videos in comparison with the handcrafted or two-dimensional (2D) CNN features-based methods.

Endoscopy is a direct imaging modality, which captures the internal structure of the human GI tract in the form of videos rather than a still image. Therefore, it is possible to extract both spatial and temporal information from endoscopic data to enhance the diagnostic capability of different deep CNN-based CAD tools. Most of the previous studies considered only the spatial information for classification and detection of different GI diseases without considering the temporal information. The loss of temporal information affects the overall performance of the CAD tools. In addition, the maximum number of classes to be managed in the previous studies were also limited to eight [9], which only considered limited GI diseases, such as a tumor or cancer.

To address these issues from previous researches, we considered 37 different categories in our proposed work, which included both normal and diseased cases related to different parts of the human GI tract. We proposed a novel two-stage deep learning-based framework to enhance the classification performance of different GI diseases by considering both spatial and temporal information. Two different models named as ResNet and LSTM were trained separately to extract the spatial and temporal features, respectively. In Table 1, the strengths and weaknesses of previous studies and our proposed method are summarized.

## 3. Contribution

This is the first approach towards the classification of multiple GI diseases that includes 37 different categories related to normal and diseased cases while considering different parts of the human GI tract. The major contributions of this study can be summarized in the following five ways when compared to the previous methods.

(1) To the best of our knowledge, this is the first approach to develop a comprehensive deep learning-based framework for the classification of multiple GI diseases by considering deep spatiotemporal features. In contrast, most of the previous studies [6,7,8,9,10,11,12,13,14,15] considered the limited number of classes that are related to a specific type of GI portion.
(2) We proposed a novel cascaded ResNet and LSTM-based framework in the medical domain to learn both spatial and temporal features for the different type of GI diseases. When compared to the previous methods based on handcrafted features and simple 2D-CNNs, our method can manage the large intraclass and low interclass variations among multiple classes more effectively.
(3) We deeply analyzed the performance of our proposed method by selecting the multilevel spatial features for LSTM from the different layers of the ResNet network. Furthermore, the performance of multilevel spatial features was also analyzed by applying principal component analysis (PCA).
(4) We compared the performance of the various state-of-the-art CNN models and different handcrafted feature-based approaches. Our analysis was more detailed, in contrast to previous studies [8,9], which provided only a limited performance analysis for a small number of classes related to a specific GI part.
(5) Finally, we have ensure that our trained model and video indices of experimental endoscopic videos are publicly available through [18]; therefore, other researchers can evaluate and compare its performance.

## 4. Proposed Method

This section presents our proposed method for the classification of multiple GI diseases, including the CNN architecture for the extraction of spatial features, LSTM-based network for the extraction of temporal features, and finally, the classification portion comprises of fully connected (FC) layers.

### 4.1. Overview of the Proposed Approach

**Figure 1:**
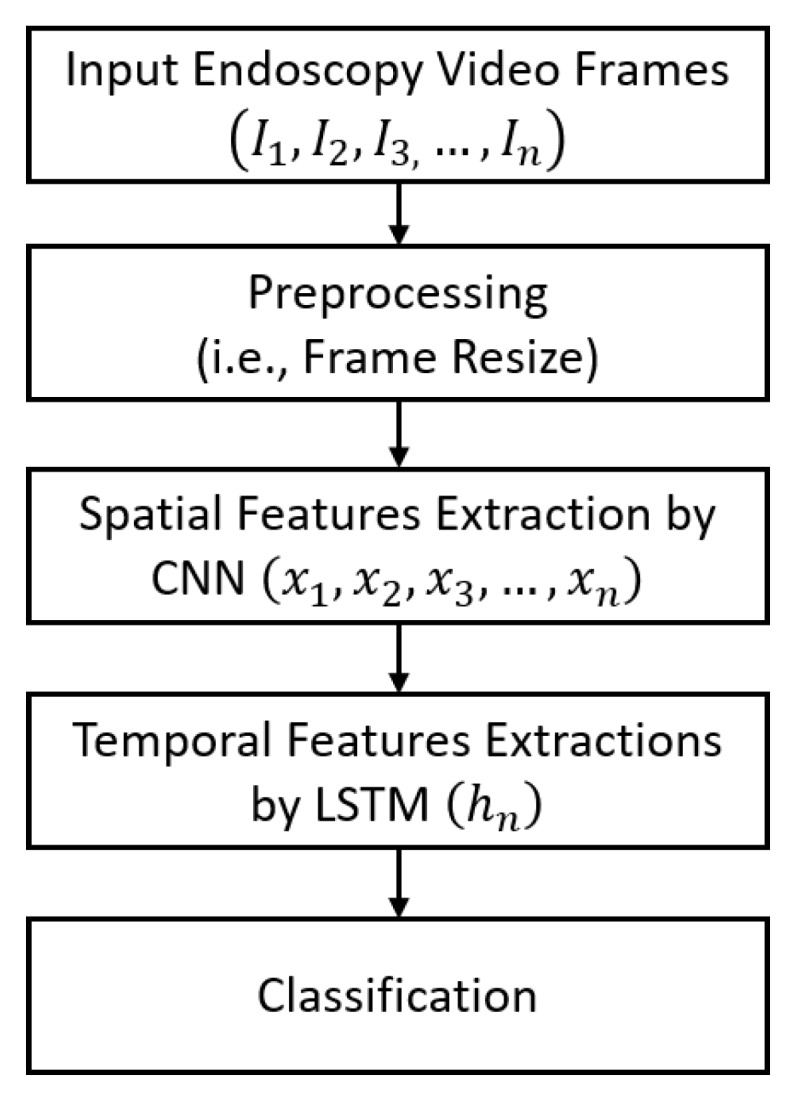
Overall flow diagram of the proposed classification framework.

The conventional image or video classification framework is comprised of two main stages, known as the feature extraction stage and the classification stage. There are also certain other preprocessing steps such as image resizing or batch normalization (BN) to adjust the dataset according to the network compatibility. A brief flowchart of our method for the classification of multiple GI diseases based on deep spatiotemporal features is illustrated in Figure 1. In the first preprocessing step, the size of each endoscopic video frame was adjusted to 224 × 224 × 3 (according to the input layer size of the CNN model). In the next steps, we used a cascaded CNN and LSTM-based deep network to extract the spatial and temporal features, respectively, by using the resized sequence of frames. Using the CNN model, a sequence of spatial feature vectors was extracted, which was subsequently inputted to the LSTM for the extraction of temporal features. The final output of the LSTM comprises of a single feature vector that contains both the spatial and temporal information for each given sequence of frames. In the last step, the classification of the extracted spatiotemporal feature vector was performed by categorizing the given video sequence into one of 37 different categories (i.e., 37 different categories presenting the normal and diseased cases related to the human GI tract).

### 4.2. Structure of Our Proposed Model

**Figure 2:**
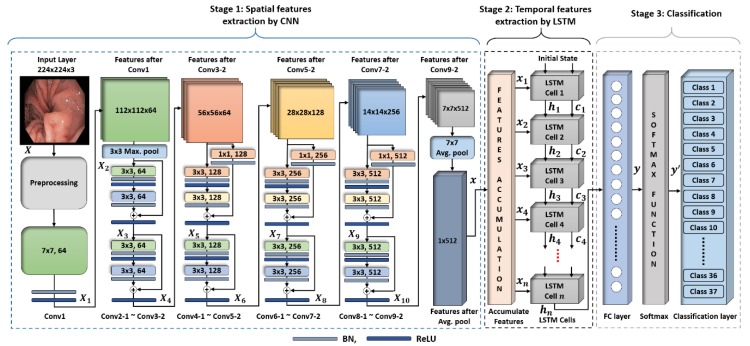
Overview of the proposed cascaded convolutional neural network and long short-term memory (LSTM)-based deep architecture for the classification of multiple gastrointestinal (GI) diseases.

Our proposed classification framework consists of a cascaded CNN and LSTM-based deep networks with the capability to classify the video data based on spatiotemporal features. The primary advantage of our network is its capability to categorize a variable length sequence of n successive images (i.e., $I_{1},I_{2},I_{3},\ldots ,I_{n}$) with significant performance gain. For example, the use of more successive images results in better classification performance. In addition, our cascaded deep learning model demonstrated high performance in comparison with only CNN-based models. That is because the CNN models only extract the spatial information by processing each input image independently rather than considering both spatial and temporal features in the case of a video dataset. Owing to the loss of temporal information in a CNN model, the overall classification performance is deteriorated. To overcome the limitation of previous spatial features-based methods in the medical domain, our study included a spatial variant of a recurrent neural network (RNN) named as LSTM along with the conventional CNN model to enhance the classification performance. The overall structure of our proposed classification framework is shown in Figure 2. The complete framework is comprised of three different stages, i.e., spatial features extraction, temporal features extraction, and finally, the classification stage. In each stage, a specific set of deep learning procedures was applied to the given input sequence of endoscopic frames. Thus, the final class label was predicted for the input sequence using 37 different categories of different GI diseases. The detailed explanation of each stage is presented in the subsequent sections.

#### 4.2.1. Spatial Features Extraction using a Convolutional Neural Network

The first stage of our proposed classification framework included a deep CNN model named ResNet18 [23], which was used for spatial features extraction from each input frame. The primary reasons for selecting ResNet18 [23] was the high classification accuracy and the optimal number of learnable parameters when compared to the other state-of-the-art deep CNN models [16,24,25,26,27]. In a later section the experimental results quantitatively illustrate the significance of our selected ResNet18 model when compared to the other models.

**Figure 3:**
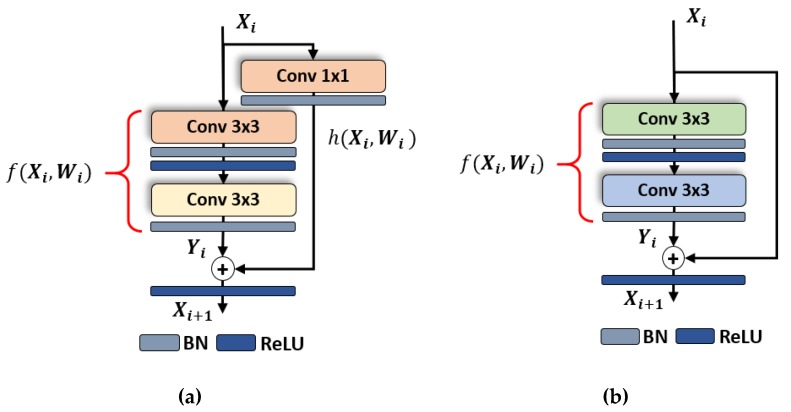
Residual block of ResNet18 with (**a**) 1 × 1 convolutional-mapping-based residual unit and (**b**) identity-mapping-based residual unit.

The complete structure of the extraction model for spatial features is illustrated in Figure 2. The entire network consists of multiple residual units, which can be considered as the basic building block. These residual units are categorized into two different types based on the type of shortcut connectivity (i.e., 1 × 1 convolutional-mapping-based shortcut connectivity and identity-mapping-based shortcut connectivity) [23]. The shortcut connectivity in an identity-mapping-based residual unit maintains the depth of previous feature map without any modification whereas the shortcut connectivity in the 1 × 1 convolutional-mapping-based residual unit increases the depth of the previous feature map by applying the 1 × 1 convolution. Moreover, in each residual unit, there are two convolutional layers with a filter size of 3 × 3 in sequential order. These filters contain the learnable parameters, which are optimized during the training procedure. ResNet18 consists of a total of eight residual units, including five identity mapping-based residual units and three 1 × 1 convolutional mapping-based residual units, as shown in Figure 3. The use of more identity mapping-based residual units results in better performance in terms of computational complexity and training time. In addition, both types of residual units result in smoother information propagation in both forward and backward directions [28].

The layer-wise structural details are further explained in Table 2, which demonstrates the flow of information processing by the different layers of ResNet18 in a sequential order. In general, the convolutional and FC layers are the main components of a conventional CNN model, which are used for features extraction and classification, respectively. There are also certain other layers without including the learnable parameters, such as a rectified linear unit (ReLU) layer, softmax, max pooling, average pooling, and a classification layer. Our selected ResNet18 model primarily contains a total of eighteen layers in which there are seventeen convolutional layers and one FC layer. These layers encompass the learnable parameters (i.e., filter coefficients and biases), which are optimized through the training procedure. Each convolutional layer is followed by the BN layer (it normalizes the feature map of each channel) and then a ReLU layer (it performs a threshold operation).

The first convolutional layer (i.e., Conv1) of our selected model generates an output feature map $X_{1}$ of size 112×112×64 by applying 64 different filters of size 7 × 7 × 3 over the given input image X. After Conv1, the next max pooling layer further processes the output feature map $X_{1}$ by applying a filter of 3×3 pixels and generates a down-sampled feature map $X_{2}$ of size 56×56×64. This output feature map $X_{2}$ is passed through the first identity mapping-based residual unit that applies the two convolution filters (Conv2-1 and Conv2-2) in sequential order and generates an intermediate feature map as $f\left(X_{2},W_{2}\right)$. Finally, the output feature map $X_{3}$ of size 56×56×64 is generated by adding $X_{2}$ and $f\left(X_{2},W_{2}\right)$. The second identity mapping-based residual unit also performs a similar operation and converts the feature map $X_{3}$ to a new feature map $X_{4}$. The next 1×1 convolutional mapping-based residual unit further processes the feature map $X_{4}$ by applying the two convolution filters (Conv4-1 and Conv4-2) in sequential order and generates the first intermediate output feature map as $f\left(X_{4},W_{4}\right)$. Meanwhile, a 1×1 convolution filter (Conv4-3) converts the feature map $X_{4}$ to the second intermediate output feature map as $h\left(X_{4},W_{4}\right)$. Finally, the output feature map $X_{5}$ is obtained by adding both intermediate feature maps $f\left(X_{4},W_{4}\right)$ and $h\left(X_{4},W_{4}\right)$.

Similarly, all the successive residual units process the output feature map of the previous residual unit in the same way by using a different number of filters with different sizes and stride values as listed in Table 2. Finally, the optimal feature vector x of size 1×1×512 is obtained after applying the average pooling layer with filter size 7×7 pixels over the last output feature map $X_{10}$ (i.e., the output of the last convolutional layer). In this way, a set of n feature vectors $\left\{x_{1},x_{2},x_{3}\ldots ,x_{n}\right\}$ are obtained by processing all the successive images ($I_{1},I_{2},I_{3}\ldots ,I_{n}$). These extracted feature vectors are further used as the input to the LSTM network for temporal feature extraction. The remaining three layers (i.e., FC, softmax, and classification layer) only participate in the training procedure. Therefore, after completing the training process, the output feature vector is selected after the average pooling layer for further temporal feature extraction and classification rather than the final classification layer.

#### 4.2.2. Temporal Features Extraction by Long Short-term Memory Model

In the second stage, LSTM, a variant of the RNN model [29], was used to exploit the temporal information from the set of n features vectors that were extracted in the first stage by using ResNet18. The structure of LSTM consists of n LSTM cells [30]. Figure 2 (Stage 2) illustrates the flow of n features vectors ($x_{1},x_{2},x_{3}\ldots ,x_{n}$) through the multiple LSTM cells. In the figure, $h_{n}$ and $c_{n}$ denote the output (also known as the hidden state) and cell state at time step n, respectively. The hidden state, $h_{n},$ contains the output of the LSTM cell for the time step n and the cell state $c_{n}$ holds the information learned from all the previous time steps (i.e., 1 to n−1). The first LSTM cell (at time step n=1) uses the initial state of the network ($h_{0},c_{0}$) and the input feature vector $x_{1}$ to compute the first output $h_{1}$ and the updated cell state $c_{1}$. At time step n (where n≠1), the LSTM cell uses the current state of the network $\left(h_{n-1},c_{n-1}\right)$ and the input feature vector $x_{n}$ to calculate the output $h_{n}$ and the updated cell state $c_{n}$. Thus, the temporal information is exploited in the LSTM stage by using all the spatial feature vectors.

**Figure 4:**
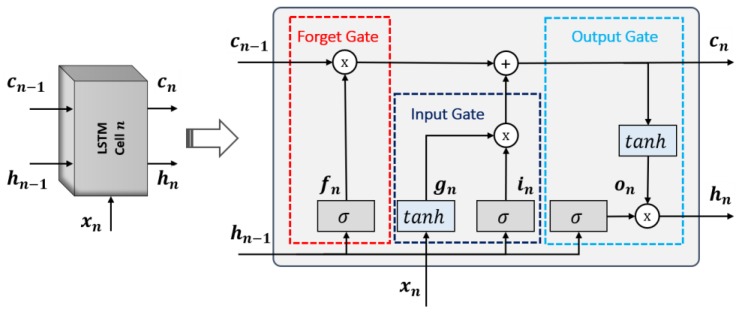
Internal connectivity of a standard LSTM cell.

The basic structure of a standard LSTM cell is shown in Figure 4, which illustrates the flow of data at time step n. In general, four components, named as input gate $\left(i_{n}\right)$, forget gate $\left(f_{n}\right)$, cell candidate $\left(g_{n}\right)$, and output gate $\left(o_{n}\right)$, are responsible for controlling the state information at time step n. The $i_{n}$ controls the level of the cell state update, whereas the $f_{n}$ controls the level of the cell state reset. The $g_{n}$ adds the information to the cell state and finally, the $o_{n}$ controls the level of the cell state added to the hidden state. Based on these components, the complete structure of the cell is divided into three gates, named as forget, input, and output gates, as highlighted in Figure 4.

Furthermore, the three different type of learnable parameters, termed as input weights, $W\;=\;{\left[W_{i_{n}},W_{f_{n}},W_{g_{n}},W_{o_{n}}\right]}^{T}$, recurrent weights, $R\;=\;{\left[R_{i_{n}},R_{f_{n}},R_{g_{n}},R_{o_{n}}\right]}^{T}$, and bias, $b\;=\;{\left[b_{i_{n}},b_{f_{n}},b_{g_{n}},b_{o_{n}}\right]}^{T}$, are included in the LSTM cell, which are responsible for learning the temporal information after performing sufficient training. These learnable parameters (W,R,b) and cell components $\left(i_{n},f_{n},g_{n},o_{n}\right)$ are used to calculate the cell state $c_{n}$ and output $h_{n}$ at time step n. The following mathematical computations are performed to determine the state information and cell components:

(1) $$c_{n}\;=\;f_{n}\times c_{n-1}+g_{n}\times i_{n}$$

(2) $$h_{n}\;=\;o_{n}\times tanh\left(c_{n}\right)$$

(3) $$i_{n}\;=\;\sigma \left(W_{i_{n}}x_{n}+R_{i_{n}}h_{n-1}+b_{i_{n}}\right)$$

(4) $$f_{n}\;=\;\sigma \left(W_{f_{n}}x_{n}+R_{f_{n}}h_{n-1}+b_{f_{n}}\right)$$

(5) $$g_{n}\;=\;tanh\left(W_{g_{n}}x_{n}+R_{g_{n}}h_{n-1}+b_{g_{n}}\right)$$

(6) $$o_{n}\;=\;\sigma \left(W_{o_{n}}x_{n}+R_{o_{n}}h_{n-1}+b_{o_{n}}\right)$$

where tanh is the hyperbolic tangent function, which is calculated as $tanh\left(x\right)=\left(e^{x}-e^{-x}\right)/\left(e^{x}+e^{-x}\right)$. It is used as a state activation function. The function σ is the sigmoid function, which is calculated as $\sigma \left(x\right)\;=\;{\left(1+e^{-x}\right)}^{-1}$ to compute the gate activation function.

In the first stage, ResNet18 processed the sequence of n successive images (i.e., $I_{1},I_{2},I_{3}\ldots ,I_{n}$) in a sequential order to extract the spatial features. Then, the LSTM model processed all the spatial feature vectors (a set of n feature vectors $\left\{x_{1},x_{2},x_{3}\ldots ,x_{n}\right\}$) in a parallel fashion in the second stage. Therefore, the feature accumulation block, as shown in Figure 2, is used to accumulate all the spatial feature vectors (obtained from ResNet18 in the first stage) before inputting it to the LSTM model in the second stage. The layer-wise structural details of our proposed LSTM model are listed in Table 3. The final output of the LSTM model contains both the spatial and temporal information, which is followed by the stack of FC layers to perform the final classification.

#### 4.2.3. Classification

In the final classification stage, the output $h_{n}$ of the LSTM cell at the last time step n is selected as the final output feature vector rather than using all the outputs (i.e., $h_{1},h_{2},h_{3},\ldots ,h_{n}$). Then, a stack consisting of FC, softmax, and classification layers is used to perform the final classification as shown in Figure 2. The output of the last LSTM cell is followed by a FC layer where the number of nodes is equal to the number of classes. The primary purpose of the FC layer is to determine the larger patterns by combining all the spatiotemporal features learned by the previous layers across the images. It multiplies the input feature vector obtained from the last LSTM cell by a weight matrix W and then adds a bias vector b. The final output obtained after this FC layer is presented as $y\;=\;W\cdot h_{n}+b$. The next softmax layer converts the output y in terms of probability by applying the softmax function [31]. Finally, the classification layer considers the output from the softmax layer and assigns each input to one of the 37 different categories by using the cross-entropy loss function [31]. In conclusion, the final class label is assigned to the given sequence of n successive images by exploiting both the spatial and temporal information.

## 5. Experimental Setup and Performance Analysis

In this section, we analyze the performance of our proposed ResNet18 and LSTM-based classification framework. We provide the details of the selected endoscopy dataset, experimental configurations, various performance analysis metrics used to evaluate the quantitative performance, observations, and analysis of the results as well as the comparison with other methods.

### 5.1. Dataset

**Figure 5:**
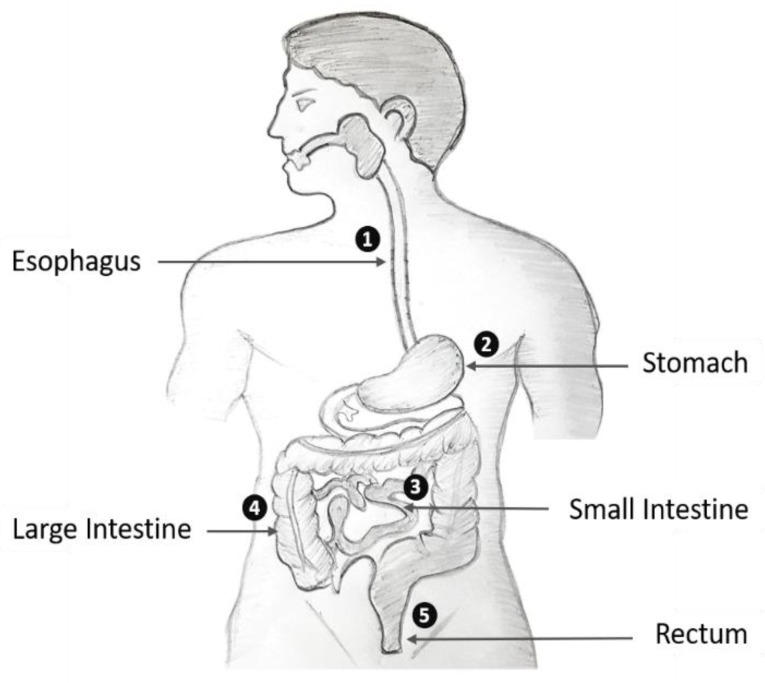
Different anatomical districts of the human GI tract.

To evaluate the performance of the proposed multiple GI diseases classification framework, we selected an open access endoscopic videos dataset from Gastrolab [32] and the KVASIR dataset [33]. The datasets contain various endoscopic videos related to different parts of the human GI tract, including both normal and diseased cases. The details of each individual video (including the information about normal and diseased cases as well as the anatomical district) are included as the video name. Based on the available information, the complete dataset was categorized into 37 different classes including both normal and diseased cases related to different parts of the human GI tract. These different classes include the multiple anatomical locations (i.e., esophagus, stomach, small intestine, large intestine, and rectum) of the human GI tract as shown in Figure 5. 

Furthermore, the details of multiple subcategories of each anatomical district and their corresponding number of classes with types of diseases and the number of training and testing sequences are listed in Table 4. The entire dataset contains a total of 77 video files including 52,471 frames. In the preprocessing part, all these frames were resized into fixed dimensions with the spatial size of 224×224; subsequently, they were converted into a standard bitmap file format. We performed the two-fold cross-validation by randomly dividing the entire dataset as 50% for training and the remaining 50% for testing. That is, in all the performance comparisons, the numbers of training data are the same as those of the testing data as shown in Table 4.

In the first stage, an online data augmentation [34] process (including random translation and in-plain rotation) was used to solve the class imbalance problem [35] caused by the different number of training samples in each class. The data augmentation process was performed only for the training dataset in the first stage (i.e., spatial features extraction using ResNet18), and was not performed for the testing dataset.

**Figure 6:**
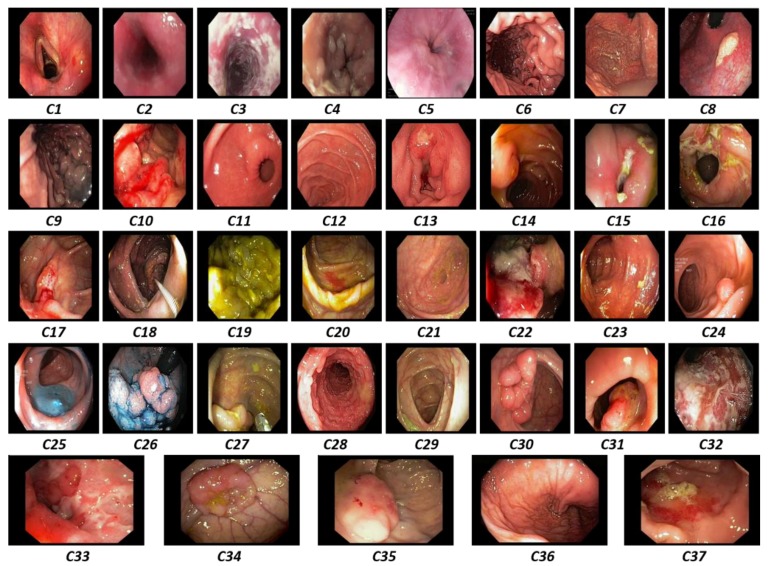
Examples from each class of the 37 different categories (i.e., ***C1*** to ***C37***) including both normal and diseased cases.

**Figure 7:**
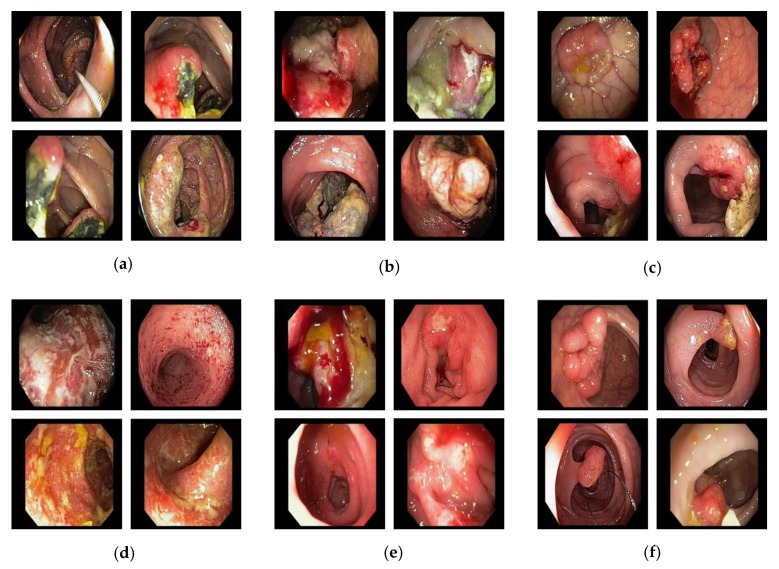
Selected sample images for illustrating the high intra-class variance: (**a**) C18; (**b**) C22; (**c**) C34; (**d**) C32; (**e**) C13; and (**f**) C30.

The visual representation of our selected dataset for each class is shown in Figure 6. In this diagram, each individual image presents a specific class from the total of 37 different classes (i.e., *C1, C2, C3, …, C37*). The primary challenge in our selected dataset was the high intra-class variance caused by the different types of lesion structures and texture properties within the same class as depicted in Figure 7. Furthermore, different viewing conditions and dynamic structural changes during the endoscopy procedure may also increase the intra-class variance. To solve this problem, a high level of abstraction was required to present the common characteristics of such types of datasets with high intra-class variance. In addition, a sufficient amount of training dataset related to a particular domain can also enhance the overall performance of the CAD systems. This type of dataset aids in analyzing the performance of our proposed framework in a challenging scenario.

### 5.2. Experimental Setup and Training

The proposed framework was implemented with MATLAB R2018b (MathWorks, Inc., Natick, MA, USA) [36] on a Windows 10 operating system. The deep learning library named as deep learning toolbox was included in MATLAB for the implementation of various CNN models [37]. Any people who purchase MATLAB R2018b [36] can use this library with the licenses based on the credits to the authors of the CNN models. All the experiments were performed on a desktop computer with a 3.50 GHz Intel® (Santa Clara, CA, USA) Core-i7-3770K central processing unit (CPU) [38], 16 GB random access memory (RAM), and an NVIDIA (Santa Clara, CA, USA) GeForce GTX 1070 graphics card [39]. The use of the graphics card provides the parallel processing capability for both the training and the testing phase.

As explained in Section 4, our proposed method combined two types of image features for classification of multiple GI diseases, i.e., the spatial features extracted by a deep CNN model in the first stage, and then the temporal features that were extracted by using the LSTM model in the second stage. Both the networks were trained separately by using the stochastic gradient descent [40] optimizer method, which is generally used for optimal training of CNNs. It is a more efficient back propagation algorithm for learning the discriminative linear classifiers by using a convex loss function. Its primary goal is to optimize the learnable parameters of the model (i.e., filter weights and biases) by considering the derivative of the loss function. In addition, we initialized the parameters of the first stage CNN model by using a pretrained ResNet18 model, which was successfully trained on the ImageNet dataset [41]. This scheme was widely used in previous studies to initialize the network parameters to make the network training process easier and time effective. In the case of the LSTM model, the initial weights were randomly initialized by using a Gaussian distribution with zero mean and 0.001 standard deviation, and the biases were initialized to zero. In Table 5, the parameters of the training procedure used in our experiments are listed.

**Figure 8:**
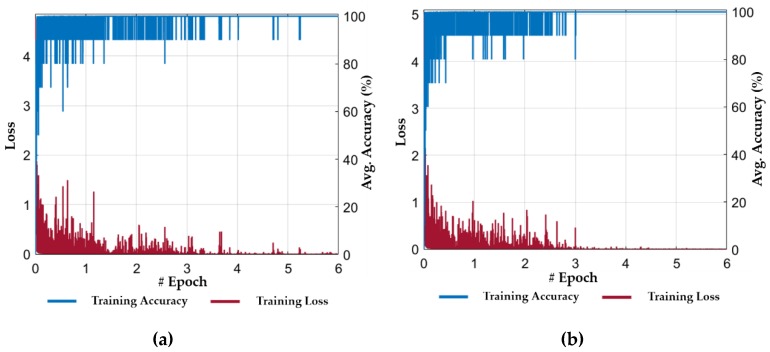
Training loss and accuracy plots during the first stage (i.e., spatial features extraction by ResNet18): (**a**) 1st fold cross-validation; and (**b**) 2nd fold cross-validation.

The performance of our proposed method was evaluated by performing the cascaded training of our ResNet18 and LSTM-based classification framework. In the first stage, we performed the training of ResNet18 by using the training dataset (as listed in Table 4). Figure 8 shows the progress of training loss and accuracy according to the different number of epochs for both folds of cross-validations. The training loss approaches zero after a certain number of epochs, and the training accuracy approaches 100%, which illustrate that our selected model is sufficiently trained. In addition, after performing several training experiments for different CNN models, we determined that the fine-tuning of a pretrained model results in faster convergence rather than training from scratch. In other words, we used the ResNet18 model which was pretrained with the ImageNet dataset [41]. Then, we performed the fine-tuning of this model with our training dataset of Table 4. Therefore, we selected a pretrained model of ResNet18 for spatial feature extraction in the first stage. Moreover, the average accuracy of our selected ResNet18 based on the spatial features was higher than other deep CNN models. Thus, both the ResNet18 and LSTM models were interconnected in a cascaded fashion, and separate trainings were performed for both networks. The second stage training process was started after completing the training for the ResNet18 model.

**Figure 9:**
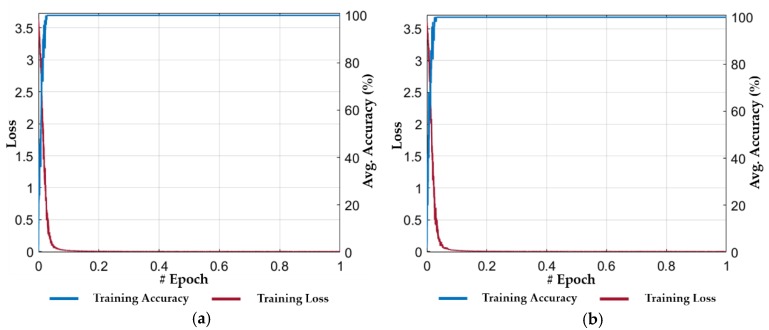
Training loss and accuracy plots during the second stage (i.e., temporal features extraction using LSTM): (**a**) 1st fold cross-validation; and (**b**) 2nd fold cross-validation.

In the second stage, the output feature vectors (extracted from the trained ResNet18 model in the first stage using the training dataset) were used to train our proposed LSTM model. In this stage, each training sample comprised of a set of n feature vectors (extracted from n successive frames in the first stage) instead of a single feature vector. Thus, an intermediate features-based dataset was generated from the extracted feature vectors, which was further used for temporal feature extraction. In our experiment, a total of fifteen (i.e., n = 15) successive frames were used to generate a set of fifteen feature vectors for each training sample. Figure 9 shows the progress of training loss and accuracy for both folds of cross-validations. The training loss approaches to zero after a certain number of iterations in the first epoch and the training accuracy approaches 100%, which shows the optimal convergence of the second stage (LSTM) of our model. In Figure 9, it can also be observed that the convergence of LSTM is faster and smoother when compared to ResNet18 (in the first stage). The primary reason for this result is the use of an intermediate dataset (i.e., a set of discriminative spatial feature vectors) for temporal feature extraction rather than using the successive frames. 

### 5.3. Evaluation of the Performance by Proposed Method

#### 5.3.1. Performance Analysis Metric

We employed average accuracy, F1 score, mean average prevision (mAP), and mean average recall (mAR) [42] to quantitatively evaluate the performance of our proposed ResNet18 and LSTM-based classification model. Based on these four parameters, we evaluated the overall performance of the model by calculating the average value for all the classes. These four metrics are defined as:

(7) $$Accuracy\;=\;\frac{1}{K}\sum_{k\;=\;1}^{K}\frac{TP_{k}+TN_{k}}{TP_{k}+TN_{k}+FP_{k}+FN_{k}}$$

(8) $$F1.Score\;=\;2\times \frac{mAP\times mAR}{mAP+mAR}$$

(9) $$mAP\;=\;\frac{1}{K}\sum_{k\;=\;1}^{K}\frac{TP_{k}}{TP_{k}+FP_{k}}$$

(10) $$mAR\;=\;\frac{1}{K}\sum_{k\;=\;1}^{K}\frac{TP_{k}}{TP_{k}+TN_{k}}$$

where $TP_{k}$, $FP_{k}$, $TN_{k}$, and $FN_{k}$ denote the number of true positives, false positives, true negatives, and false negatives, respectively, for each class k. The value of $TP_{k}$ presents the number of correctly classified images from class k, $FP_{k}$ shows the number of images that are misclassified as belonging to class k. $TN_{k}$ indicates the number of images correctly classified that do not belong to class k and $FN_{k}$ denotes the number of misclassified images that actually belong to class k. Here K denotes the total number of classes, which is equal to 37 in our research.

#### 5.3.2. Testing of the Proposed Method

**Figure 10:**
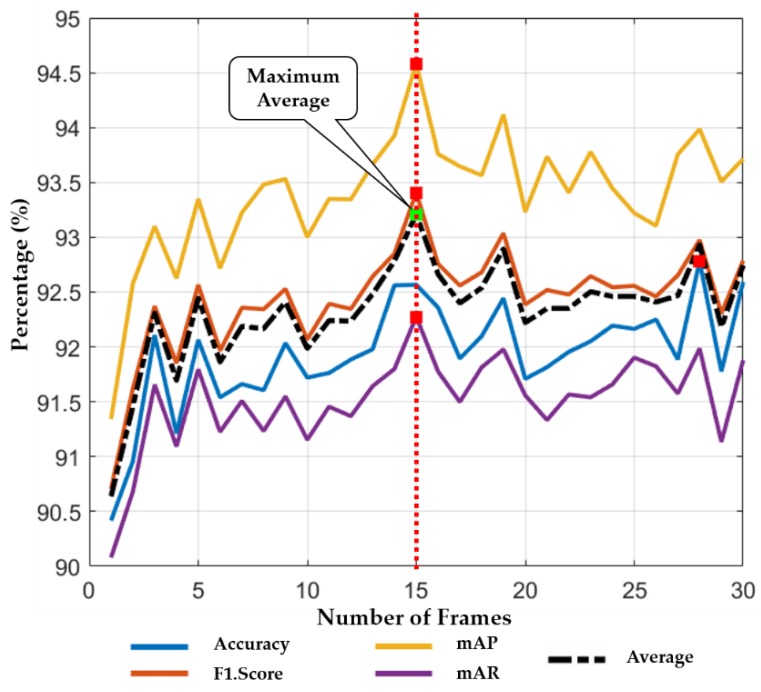
Classification performance of our framework according to the number of frames (n) for LSTM.

The length of successive frames performs an important role in the system performance. The small number of successive frames results in low temporal information, whereas the long sequence length increases the processing time and the effects of noise. Therefore, we performed the training of our LSTM model for thirty different number of frames (i.e., n = 1,2,3,…,30). Then, the testing performance was evaluated for each step size. Figure 10 shows the average performance results according to different number of frames. In Figure 10, the green square box indicates the maximum average performance whereas the red square box illustrates the maximum performance with respect to different performance metrics (i.e., accuracy, F1 score, mAP, and mAR). Finally, based on the overall maximum average performance, we determined that the best accuracy could be obtained when the numbers of frame was 15 (n = 15).

As the next experiment, we performed a layer-wise performance comparison between our method (ResNet18 + LSTM) and only a ResNet18 model by selecting the features from the different parts of the network. Moreover, this additional experiment was also used to investigate the more discriminative features at certain intermediate layers that could result in better performance. For this experiment, the output feature vectors were extracted from five different layers (i.e., Conv6-2, Conv7-2, Conv8-2, Conv9-2, Avg. pooling, as listed in Table 2) of ResNet18 with the feature map size of 14 × 14 × 256 (50,176), 14 × 14 × 256 (50,176), 7 × 7 × 512 (25,088), 7 × 7 × 512 (25,088), and 1 × 1 × 512 (512), respectively. In the case of our method, the classification performance for each layer was obtained by further extracting the temporal information from the LSTM model using these features. The layer-wise features from ResNet18 model were classified using a k-nearest neighbor (KNN) classifier, which is widely used for pattern classification [43]. The complete layer-wise performances of our method and ResNet18 are listed in Table 6. Based on the overall performance, we concluded that the deeper features result in better classification performance in the case of our method and the ResNet18 model. However, the layer-wise performance of our method was still higher than the conventional ResNet18.

Moreover, our method also showed a high average accuracy of 90.48% and mAP of 91.29% when a still image (i.e., n = 1) was used, which are higher values when compared to other CNN-based methods (an accuracy of 89.95% and mAP of 90.72% in the case of conventional ResNet18).

The extracted features from the last average pooling layer of ResNet18 were further analyzed by applying PCA [44] technique as a post processing step. The main objective of this analysis was to explore the discriminative nature of the features (i.e., to check if our selected features were distinctive or redundant). For this purpose, all the extracted features of dimension 1×512 from the last average pooling layer were projected to the eigenspace by applying the PCA. This eigenspace presented all the input feature vectors in a new coordinate system in a more distinctive way. The dimensions of these newly obtained features are selected based on the maximum variance (i.e., greater than 99%) of the projected data on all the possible axes. The eigenvalue corresponding to each feature vector was used to select a feature vector. In the case of our dataset, a new set of feature vectors (with the feature dimension 1×136) was obtained by selecting a total of 136 eigenvectors with the highest eigenvalues. In our proposed model, this new set of feature vectors were further used as inputs to the LSTM model to explore the temporal information and then the final classification performance was obtained as listed in Table 7. In addition, the PCA feature-based performance was evaluated for ResNet18 by using the KNN classifier, which is also presented in Table 7. According to these final classification results, we concluded that the PCA-based features reduced the performance in both cases (i.e., our proposed model and ResNet18), whereas the original high dimension features resulted in better performance. Finally, it can be concluded that our extracted features (from the last average pooling layer) were already diverse, and the performance of our method was still high in comparison with conventional ResNet18 after applying the PCA.

**Figure 11:**
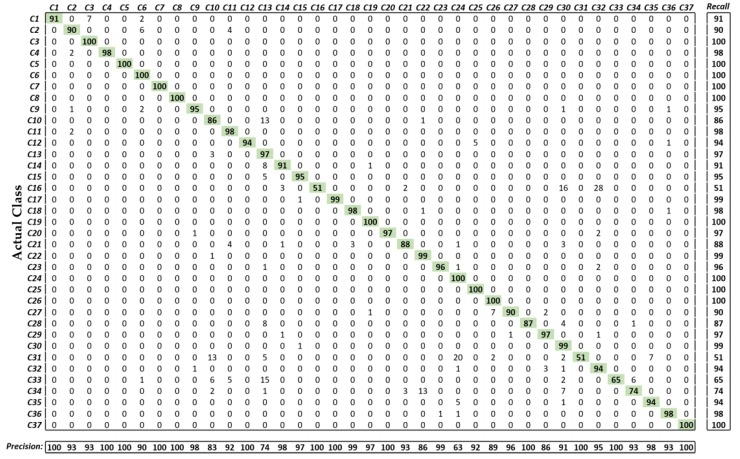
Confusion matrix of the proposed method. The entry in the ith row and jth column corresponds to the percentage of samples from class i that were classified as class j. Precision and recall are calculated as “*TP_k_*/ (*TP_k_* + *FP_k_*) “and “*TP_k_*/ (*TP_k_* + *FN_k_*)” [45], respectively.

Figure 11 illustrates the more comprehensive classification performance of our model in terms of the confusion matrix. It can be observed from these results that only a few classes (i.e., *C16*, *C31*, *C33*, *C34*) showed a low classification performance owing to the high inter class similarities in terms of lesion textures or GI organ structures. However, the overall performance of our proposed method was significantly high for a dataset with several classes.

#### 5.3.3. Comparisons with Previous Methods

The performance of our proposed ResNet18 and LSTM-based methods were compared with the various state-of-the-art deep CNN-based CAD tools that are used in the endoscopy domain [8,12,14,15]. To ensure a fair comparison, the performances of all the existing baseline methods were evaluated with our selected dataset using the same training and testing data of two-fold cross-validation. In a recent study related to endoscopy, two different CNN models—GoogLeNet [8,12,15] and InceptionV3 [14]—were primarily used in the diagnosis of various type of GI diseases. Therefore, the performance of these two models were evaluated in comparison with our proposed method. The experimental results showed that our method outperformed these two deep CNN models [8,12,14,15] with significant performance gain as listed in Table 8.

Further, we also compared the performance of our method with the recent CNN models [16,23,24,25] used in image classification domains other than endoscopy. The main objective of these comparisons was to estimate the performance of the existing state-of-the-art CNN models in the endoscopy image analysis domain. The complete experimental results for all the selected baseline methods are listed in Table 8. These results confirm that our proposed ResNet18 and LSTM-based method shows the highest performance in the endoscopy image analysis domain for the classification of multiple GI diseases.

**Figure 12:**
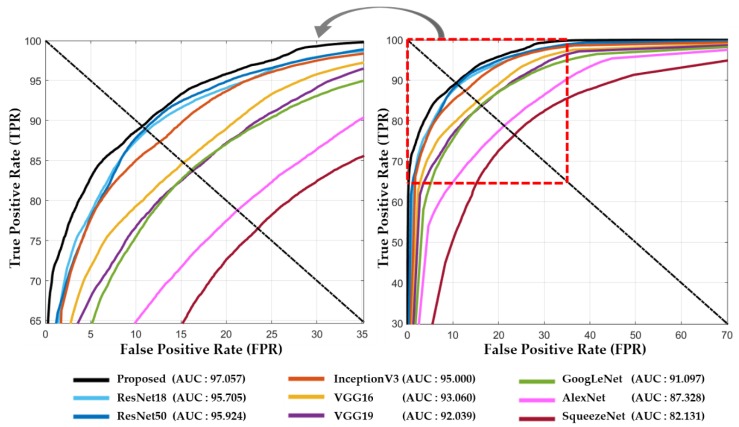
Receiver operating characteristic curves of our proposed method and other baseline models with the area under the curve (AUC).

The discriminative ability of our proposed method, in contrast with other baseline methods, can also be observed through the receiver operating characteristics (ROC) curve (an effective measure used to evaluate the diagnostic ability of a model). It is created by plotting the true positive rate (known as the probability of detection) against the false positive rate (known as the probability of false alarm) at various threshold settings. From Figure 12, it can be observed that our proposed method also shows the highest value for the area under the curve (AUC) with a value of 97.057% in comparison with all the other selected baseline methods (i.e., SqueezeNet: 82.131%, AlexNet: 87.328%, GoogLeNet: 91.097%, VGG19: 92.039%, VGG16: 93.060%, InceptionV3: 95.000%, ResNet50: 95.924%, and ResNet18: 95.705%). All these ROC curves are presented by the average values obtained from two-fold cross-validations. The figure on the left side provides an enlarged view to illustrate the performance difference more clearly.

The complete parametric and structural details of our proposed model and the other selected models are listed in Table 9. The AUC performance of ResNet18 is comparable with the second-best model named as ResNet50, as shown in Figure 12; however, the training parameters of ResNet18 are significantly less than half of that of ResNet50, as listed in Table 9. Therefore, we adopted the ResNet18 architecture as the backbone model to extract the spatial features, which are further used as inputs to the LSTM model to exploit the temporal information. In our proposed framework, the total learnable parameters were approximately 13.17M (including both ResNet18 and LSTM), which were still significantly lower than the second-best model (i.e., ResNet50) as shown in Table 9.

**Figure 13:**
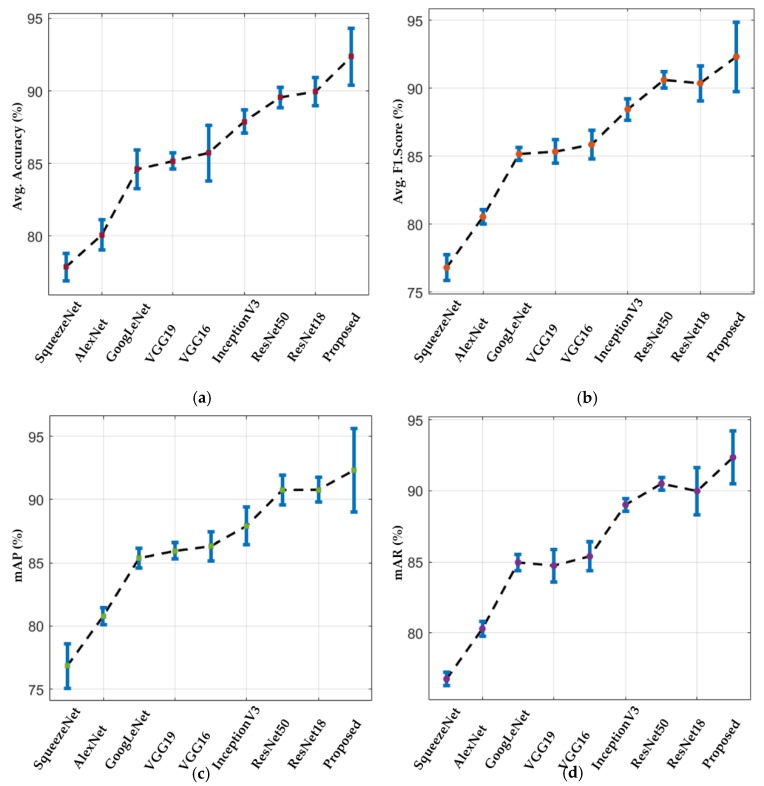
Sensitivity analysis plot of our method and various baseline models in terms of (**a**) average accuracy; (**b**) average F1 score; (**c**) mAP; and (**d**) mAR.

Furthermore, a sensitivity analysis was performed to evaluate the robustness of our method and other CNN models. A Monte Carlo simulation step [27] was performed to analyze this sensitivity performance. In this simulation setup, the performance of each individual CNN model was evaluated in an iterative way by randomly selecting 20% of the complete testing dataset as a subset of the testing dataset. A total of 200 iterations were performed for both folds of cross-validations. Finally, the average performance (i.e., average accuracy, F1 score, mAP, and mAR) as well as standard deviation were obtained for each model. The overall sensitivity performance of our method and all the selected models are illustrated in Figure 13. It can be observed in Figure 13a–d that the overall sensitivity performance of our proposed method is higher while considering average accuracy, F1 score, mAP, and mAR when compared to all the existing baseline models.

**Figure 14:**
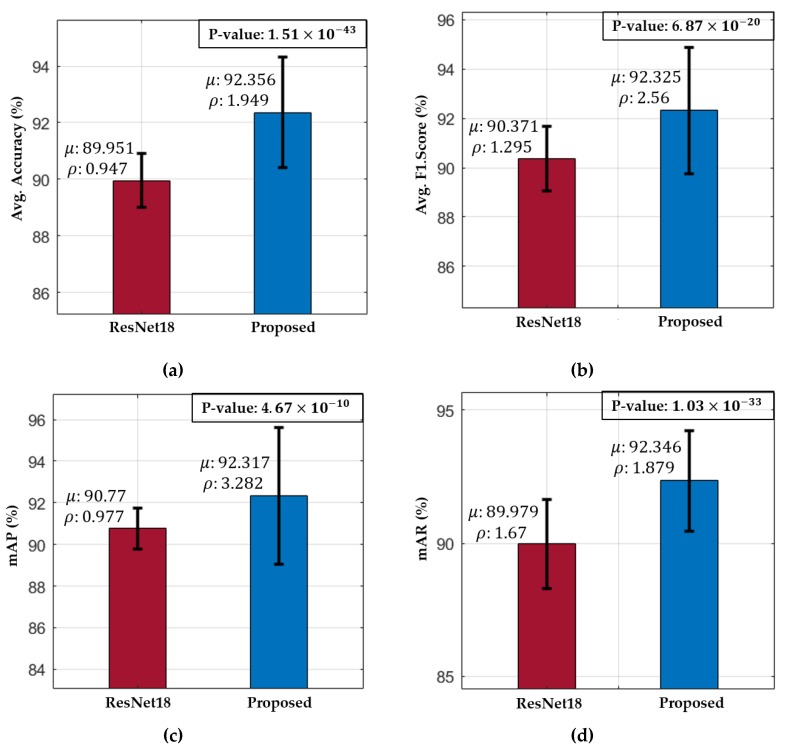
t-test performance of our method and the second-best model in terms of (**a**) average accuracy; (**b**) average F1 score; (**c**) mAP; and (**d**) mAR.

A t-test performance analysis [46] was further performed to illustrate the significance of the performance difference between our method and ResNet18. The reason why the t-test performance analysis was performed only against ResNet18 is because ResNet18 shows the second-best accuracy as shown in Table 8. In general, this performance analysis is often used to illustrate the performance difference between two systems or algorithms in a more discriminative way. It is based on a null hypothesis (H), which assumes that there is no performance difference (i.e., H = 0) between two models. Then, a rejection score (*p*-value) is calculated to check the validity of the null hypothesis based on the performance of the two models (in this case, our method and the second-best model). Figure 14 illustrates the *t*-test performance (for the values of mean (μ), standard deviation (ρ), and *p*-value) for our method and the second-best model. These results were calculated for all the performance measures. The obtained rejection scores (p-values) in case of the average accuracy, F1 score, mAP, and mAR were $1.51\times 10^{-43}$, $6.87\times 10^{-20}$, $4.67\times 10^{-10}$, and $1.03\times 10^{-33}$, respectively. All these *p*-values are less than 0.01, which indicate that the null hypothesis is rejected (i.e., H≠0) at a 99% confidence score for all the performance metrics. Based on these results, it can be concluded that there is a significant performance difference between our method and the second-best method. Furthermore, the higher mean (μ) performance of our method indicates its superiority over the second-best baseline model. 

We also performed Cohen’s d [47] analysis, by which the size of the difference between the two groups were demonstrated using the effect size [48]. Cohen’s d analysis is widely used for analyzing the difference between two measured values. Generally, Cohen’s d is classified as small at approximately 0.2–0.3, as medium at approximately 0.5, and as large at greater than or equal to 0.8. For example, if the calculated Cohen’s d is closer to 0.2–0.3 than 0.5 and 0.8, we can say that the difference between measured values has a small effect size. If the calculated Cohen’s d is closer to 0.8 than 0.2–0.3 and 0.5, we can say that the difference between measured values has a large effect size. The calculated Cohen’s d values for the performance of the two models (our method and the second-best model) were approximately 1.57 (closer to 0.8), 0.96 (closer to 0.8), 0.64 (closer to 0.8), and 1.33 (closer to 0.8) for average accuracy, F1 score, mAP, and mAR, respectively. Consequently, we concluded that the difference in the performances between our method and the second-best model has a large effect while considering the average accuracy, F1 score, mAP, and mAR.

In this section, we present the performances of various handcrafted feature-based methods that were also compared with our proposed CNN and LSTM-based classification framework for further comparison. In this comparison, three known handcrafted feature extraction methods, named as LBP [49], histogram of oriented gradients (HoG) [50], and multilevel LBP (MLBP) [51], were considered. Then, the extracted features from each method were classified by using four different classifiers: adaptive boosting (AdaBoostM2) [52], multiclass SVM (multi-SVM) [53], random forest (RF) [54], and KNN. All these handcrafted feature-based methods exploit the low-level features (i.e., edge or corner information). We evaluated the performance of 12 different handcrafted feature-based classification methods for our selected dataset to obtain a fair comparison. The detailed results for all these classification methods are listed in Table 10.

Among all these handcrafted feature extraction and classification methods, HoG + RF (i.e., HoG feature extraction method followed by the RF classifier) demonstrated superior performance. Hence, the HoG feature extraction method exploited the more discriminative low-level features in comparison with the other two methods. Furthermore, the RF classifier considers a tree structure to determine the classification decision, which resulted in a better performance and controlled the over-fitting problem. However, there is a significant performance difference between our method and the best handcrafted feature-based method (HoG + RF). Our proposed method outperformed all the handcrafted feature-based methods.

## 6. Discussion

**Figure 15:**
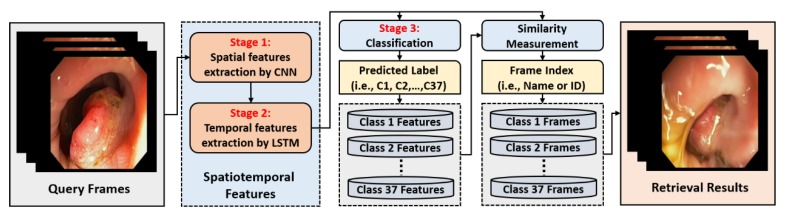
Class prediction-based retrieval system by using our proposed classification framework.

Our proposed deep CNN and LSTM-based classification framework shows the best performance with a high AUC of 97.057%. This remarkable performance of our proposed system increases its usability in the diagnosis of several GI diseases by automatically detecting different types of GI lesions or abnormalities, such as polyps, ulcers, or cancers from endoscopic videos. Our AI-based CAD system can assist the physicians in an effective diagnosis and treatment of many complex GI diseases. Furthermore, the classification of the endoscopic videos can, itself, be beneficial in retrieving the previously stored videos related to the current situation of a patient. Thus, the past cases can provide a path toward correct diagnostic decision. Therefore, we can also utilize our proposed classification framework for efficient endoscopic video frame retrieval by using the predicted class labels. The overall block diagram for our class prediction-based retrieval system is shown in Figure 15. In this retrieval section, the first step is to predict the actual class for the given query (i.e., successive endoscopic video frames). To predict the actual class label, a probability score corresponding to each class label is obtained for the given query by using our proposed classification framework. Based on the highest probability score, the corresponding class label is chosen as the actual class label. In the second step, the relevant cases related to input query frames are explored only within the predicted class based on feature matching. In this feature matching stage, the extracted spatiotemporal feature vector from the input query frames is matched one by one with the feature database of that predicted class by calculating the Euclidean distance. Based on the minimum distance, the frame index (i.e., name or ID information) is selected. Finally, the relevant frame is retrieved from the database by using the frame index information obtained in previous stage.

**Figure 16:**
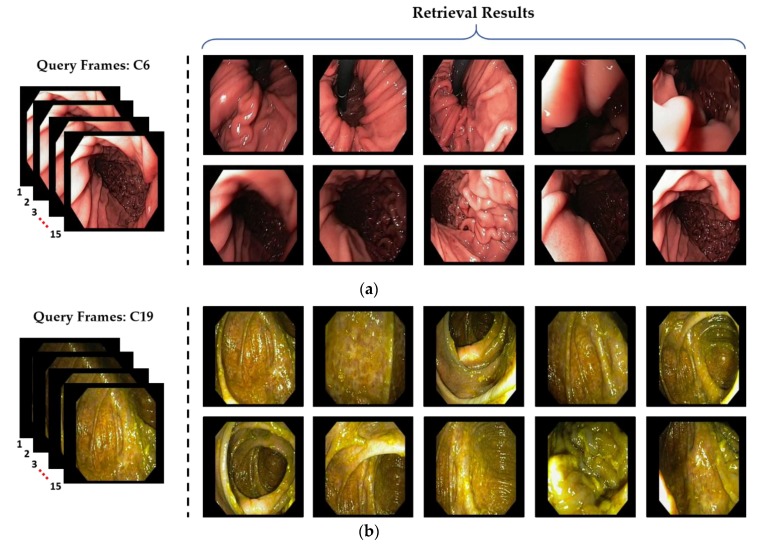

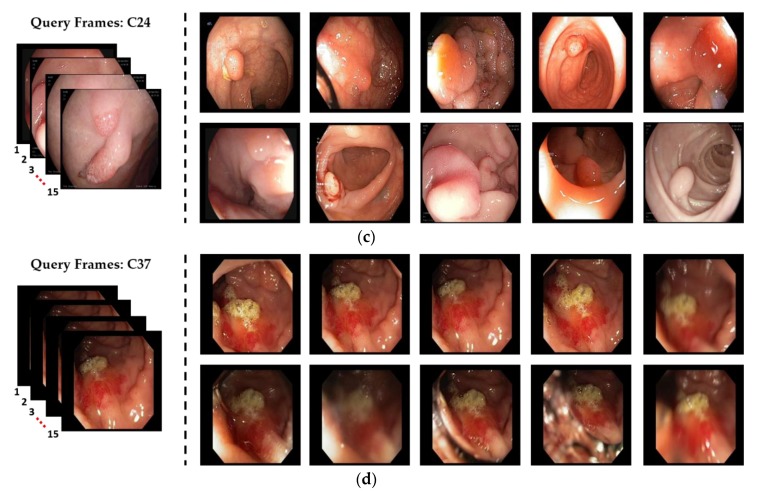
Examples of the correctly retrieved frames by our proposed method: (**a**) C6; (**b**) C19; (**c**) C24; and (**d**) C37.

**Figure 17:**
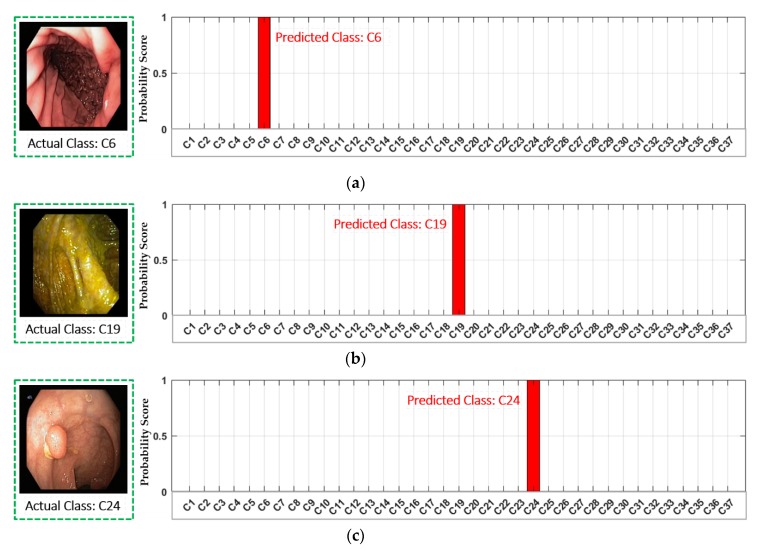

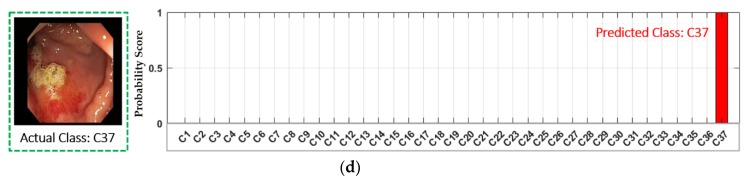
Examples of the correctly classified frames by our proposed method with probability score graph: (**a**) C6; (**b**) C19; (**c**) C24; and (**d**) C37.

A few correctly retrieved examples are illustrated in Figure 16 by using our class prediction-based retrieval system. It can be observed that the retrieved endoscopic frames have high intra-class variance with varying illumination and contrast. However, our proposed system still outperforms with 100% retrieval performance for all the selected cases. Moreover, the classification performance for these selected example cases is also 100%, which can be observed in Figure 11 (confusion matrix performance for each class). Further, Figure 17 shows the probability score corresponding to each input query. It can be observed that the highest probability score is obtained for the actual predicted class, which shows that the proposed classification model is capable of extracting the discriminative features for the given query. In conclusion, this significant performance gain (in both classification and retrieval sections) shows that our method can be robust to the high intra-class variance of a dataset.

**Figure 18:**
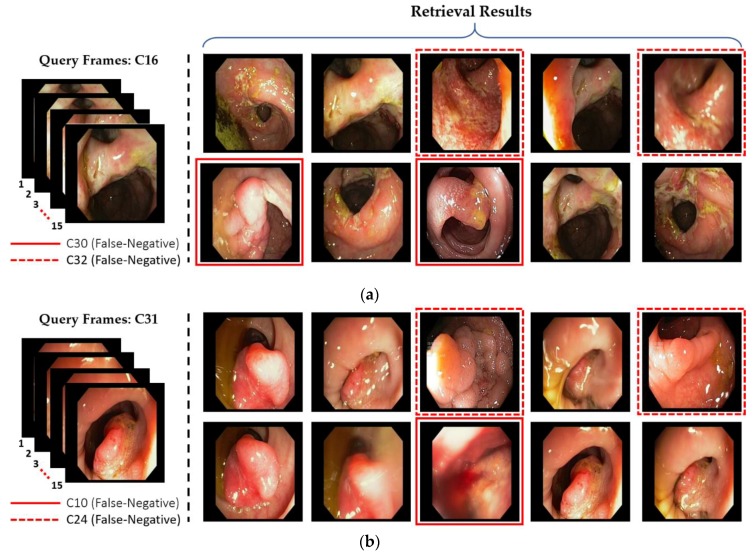

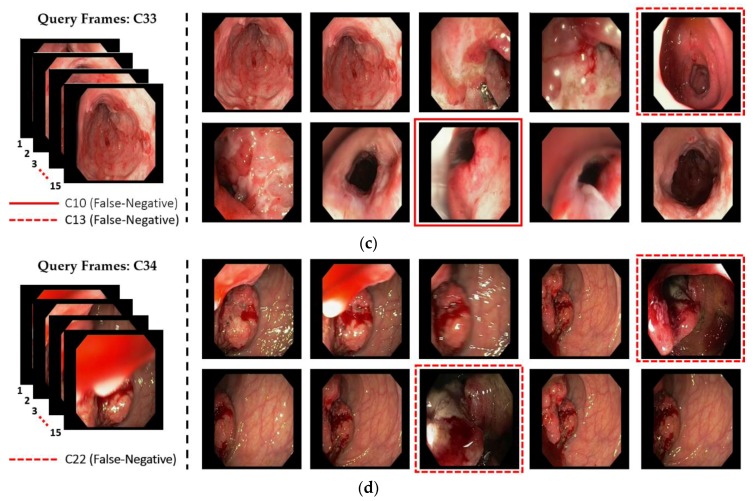
Examples of the incorrectly retrieved frames by our proposed method: (**a**) C16; (**b**) C31; (**c**) C33; and (**d**) C34.

**Figure 19:**
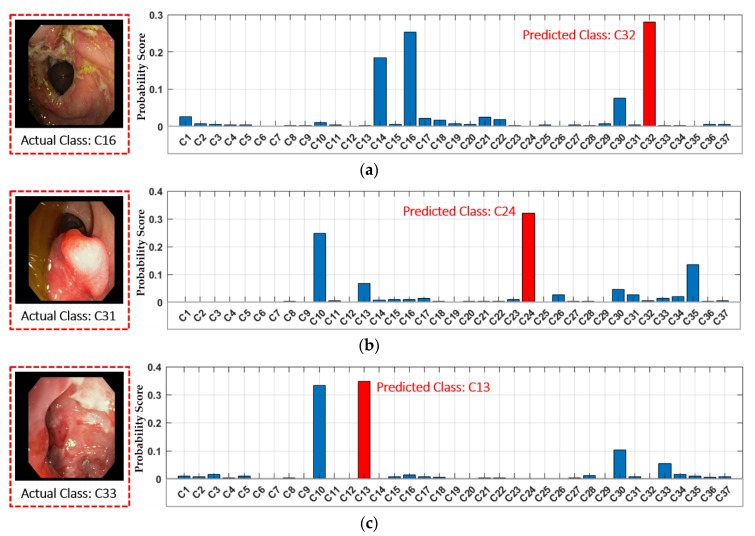

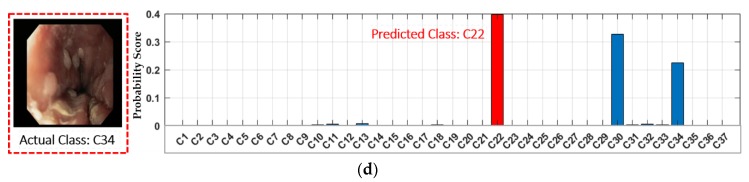
Examples of the incorrectly classified frames by our proposed method with probability score graph: (**a**) C16; (**b**) C31; (**c**) C33; and (**d**) C34.

There are a few classes in our selected dataset that show the low retrieval performance, as shown in Figure 18. The primary reason for this performance degradation is the anatomical structural overlapping and identical shape of different GI lesions among different classes. Figure 18a shows a few incorrectly retrieved results as C30 (i.e., tuber adenoma in sigmoid colon) and C32 (i.e., ulcerative colitis in rectosigmoid part of large intestine) are retrieved for an input query of C16 (i.e., severe Crohn’s disease in terminal ileum of small intestine). It can be observed from Figure 18a that the lesion characteristics among these three classes (i.e., C16, C30, and C32) show a resemblance that may cause the incorrect retrieval. Similarly, certain other incorrect retrieval cases were obtained for an input query of C31 (i.e., polypoid cancer in sigmoid colon), C33 (i.e., severe Crohn’s disease in the rectum), and C34 (i.e., adenocarcinoma in the rectum) owing to identical lesion characteristics, as shown in Figure 18b–d. Moreover, Figure 19 shows the probability score corresponding to each input query in which significantly higher probability scores can be observed corresponding to multiple predicted class labels. These multiple higher scores show the structural or lesion similarities among multiple classes, which can result in classification errors. However, the retrieval performance in these cases can be enhanced by exploring the input query in multiple classes, which can be selected based on a multiple probability scores that is greater than a certain threshold.

## 7. Conclusions

In this paper, a novel CNN and LSTM-based classification framework was proposed for the classification of multiple GI diseases using endoscopic videos. Moreover, our proposed classification framework is further utilized to design a class prediction-based endoscopic video retrieval system. The proposed spatiotemporal features-based method is capable of encoding more discriminative representations of multiple endoscopy scans when compared to the features learned only from spatial information. Therefore, both spatial and temporal information results in better classification and retrieval performance. The performance of the proposed method was evaluated thoroughly using a publicly available dataset from GastroLab as well as the KVASIR database. Moreover, the same dataset and experimental protocol was adopted for the various state-of-the-art methods to make a fair comparison. The proposed method achieved 97.057% area under the curve as the best results, together with an average accuracy of 92.57%, F1 score of 93.41%, mAP of 94.58%, and mAR of 92.28. In addition, the obtained t-test rejection scores (*p*-values) of our proposed and second-best method are less than 0.01 ($1.51\times 10^{-43}$, $6.87\times 10^{-20}$, $4.67\times 10^{-10}$, and in the case of the average accuracy, F1 score, mAP, and mAR, respectively), which indicate that the null hypothesis is rejected (i.e., H≠0) at a 99% confidence score for all the performance metrics. After performing a detailed analysis, we observed that our method consistently achieved high classification performance in comparison with various state-of-the-art deep CNN and handcrafted features-based methods of LBP, HoG, and MLBP. The classification and retrieval performance of the proposed system reveals its applicability to clinical diagnosis, treatment, education, and research. We also ensured that our trained model is publicly available to aid other researchers in performance comparisons.

As a future work, we are planning to increase the dataset by considering more than 37 classes. In addition, we are planning to perform the real-time detection of small lesions using an endoscopic video. We also plan to improve the overall classification performance by combing multiple deep CNN models.

## Figures and Tables

**Table 1:** Comparison of our proposed and existing methods for endoscopy disease classification.

Endoscopy Type	Method	Purpose	No. of Classes	Strength	Weakness
CE	Log Gabor filter, SUSAN edge detection and SVM [19]	Small bowel polyps and ulcers detection	2	Computationally efficient	Limited dataset and number of classes Low detection performance
CE	Texture features (ULBP, wavelet) + SVM [20]	Polyp detection in GI tract	2	Robust to illumination change and scale invariant	Limited dataset and number of classes
CE	Texture features (LBP, wavelet) + SVM [6]	Tumor recognition in the digestive tract	2	Invariant to illumination change Extract multiresolution features	Limited dataset and number of classes
CE	Texture features (SIFT, Saliency) + SVM [21]	Polyp classification	2	Extract scale invariant features	Limited dataset and number of classes
CE	Texture features (SIFT, HoG, LBP, CLBP, ULBP) + SVM, FLDA [22]	Polyp Detection	2	Extract scale invariant features High classification performance	Limited dataset and number of classes
CE	CNN [7]	Small intestine movement characterization	6	High classification performance	Limited number of classes
CE	CNN [15]	Celiac disease classification	2	High sensitivity and specificity	Limited dataset and number of classes
CE	CNN [12]	Hookworm detection	2	Edge extraction network results in better performance	Limited number of classes
EGD	CNN [9]	*H. pylori* infection detection	9	Comparable performance of second CNN with the clinical diagnosis reference standard	CAD performance should be enhanced. A limited number of classes
EGD	CNN [8]	Anatomical classification of GI images	6	High classification performance Computationally efficient	Limited number of classes Only used for anatomical classification
EGD	CNN-based SSD detector [13]	Gastric cancer detection	2	High sensitivity Computationally efficient	Overall low positive prediction value Limited dataset and number of classes
Colonoscopy	CNN [10]	Colorectal polyp detection and classification	3	High detection performance	Limited dataset and number of classes Low classification performance
Colonoscopy	CNN [14]	Real-time colorectal polyp type analysis	4	High accuracy and sensitivity	Limited number of classes Low specificity
Colonoscopy	Online and offline 3D-CNN [11]	Detection of colorectal polyps	2	Computationally efficient	CAD performance should be enhanced.
EGD, Colonoscopy, Sigmoidoscopy, Rectoscopy	CNN (ResNet) + LSTM (Proposed)	Classification of multiple GI diseases	37	Computationally efficient High classification performance	Cascaded training of CNN and LSTM requires more time

**Table 2:** Layer-wise configuration details of deep ResnNet18 model in our study.

Layer Name	Feature Map Size	Filters	Kernel Size	Stride	#Padding	Total Learnable Parameters
Image input layer	224×224 ×3	n/a	n/a	n/a	n/a	n/a
Conv1	112×112×64	64	7×7×3	2	3	9600
Max pooling	56×56×64	1	3×3	2	1	
Conv2-1–Conv2-2 (Identity Mapping)	56×56×64 56×56×64	64 64	3×3×64 3×3×64	1 1	1 1	74,112
Conv3-1–Conv3-2 (Identity Mapping)	56×56×64 56×56×64	64 64	3×3×64 3×3×64	1 1	1 1	74,112
Conv4-1–Conv4-2 (1 × 1 Convolutional Mapping)	28×28×128 28×28×128 28×28×128	128 128 128	3×3×64 3×3×128 1×1×64	2 1 2	1 1 0	230,528
Conv5-1–Conv5-2 (Identity Mapping)	28×28×128 28×28×128	128 128	3×3×128 3×3×128	1 1	1 1	295,680
Conv6-1–Conv6-3 (1 × 1 Convolutional Mapping)	14×14×256 14×14×256 14×14×256	256 256 256	3×3×128 3×3×256 1×1×128	2 1 2	1 1 0	919,808
Conv7-1–Conv7-2 (Identity Mapping)	14×14×256 14×14×256	256 256	3×3×256 3×3×256	1 1	1 1	1,181,184
Conv8-1–Conv8-3 (1 × 1 Convolutional Mapping)	7×7×512 7×7×512 7×7×512	512 512 512	3×3×256 3×3×512 1×1×256	2 1 2	1 1 0	3,674,624
Conv9-1–Conv9-2 (Identity Mapping)	7×7×512 7×7×512	512 512	3×3×512 3×3×512	1 1	1 1	4,721,664
Avg pooling	1×1×512	1	7×7	7	0	
FC layer	37					18,981
Softmax	37					
Classification layer	37					
Total number of learnable parameters: 11,200,293						

**Table 3:** Layer-wise configuration details of long short-term memory (LSTM) model in our study.

Layer Name	Feature Map Size	Total Learnable
Sequence input layer	n×1×1×512	
LSTM	600	1,951,200
Dropout	600	
FC layer	37	22,237
Softmax	37	
Classification layer	37	
Total learnable parameters: 1,973,437		

**Table 4:** Details of multiple subcategories of each anatomical district and their corresponding classes.

Gastrointestinal Tract		Class Name (Normal/Disease Cases)	Training Set (Frames)	Testing Set (Frames)	Total
Anatomical District	Subcategory				
**Esophagus**	Larynx	C1: Normal	387	387	774
	Upper part	C2: Normal	625	625	1250
		C3: Esophageal candidiasis	419	419	838
		C4: Esophageal papillomatosis	272	272	544
	Lower part (z-line)	C5: Normal	250	250	500
**Stomach**	Cardia	C6: Hiatal hernia	648	648	1296
	Fundus	C7: Atrophic gastritis	241	241	482
		C8: Atrophic and xanthoma gastritis	255	254	509
	Body	C9: Benign hyperplastic polyps	1070	1070	2140
		C10: Adenocarcinoma (Cancer)	955	955	1910
	Pylorus	C11: Normal	1275	1275	2550
**Small Intestine**	Duodenum	C12: Normal	423	423	846
		C13: Ulcer	1345	1345	2690
		C14: Papilla vateri	702	702	1404
	Terminal Ileum	C15: Crohn’s disease	840	840	1680
	Ileocecal	C16: Severe Crohn’s disease	278	278	556
	Ileocecal valve	C17: Crohn’s disease	838	838	1676
**Large Intestine**	Caecum	C18: Adenocarcinoma (Cancer)	1301	1301	2602
		C19: Melanosis coli	342	342	684
		C20: Caecal angiectasia	403	404	807
		C21: Appendix aperture	694	694	1388
	Ascending/ Transverse/Descending Colon	C22: Adenocarcinoma (Cancer)	1293	1293	2586
		C23: Melanosis coli	603	604	1207
		C24: Other types of polyps	250	250	500
		C25: Dyed resection margins	250	250	500
		C26: Dyed lifted polyps	250	250	500
		C27: Melanosis coli and tuber adenoma	243	243	486
		C28: Inflammatory polyposis	382	382	764
		C29: Normal	500	500	1000
	Sigmoid Colon	C30: Tuber adenoma	2212	2212	4424
		C31: Polypoid cancer	282	282	564
	Rectosigmoid	C32: Ulcerative colitis	2071	2071	4142
**Rectum**		C33: Severe Crohn’s disease	1074	1074	2148
		C34: Adenocarcinoma (Cancer)	1362	1362	2724
		C35: Tuber adenoma	1069	1069	2138
		C36: Normal	420	420	840
		C37: A focal radiation injury	411	411	822

**Table 5:** Parameters of the stochastic gradient descent method for the training of both ResNet18 and LSTM models in our experiments.

Model	Number of Training Epochs	Initial Learning Rate	Momentum	L2-Regularization	Learning Rate Drop Factor	Mini-Batch Size
ResNet18	8	0.001	0.9	0.0001	0.1	10
LSTM	10	0.0001	0.9	0.0001	0.1	50

**Table 6:** Performance comparison of our method using ResNet18 + LSTM with the conventional ResNet18 model based on feature extraction from different layers (unit: %).

Layer Name	Feature Dim.	ResNet18 [23]				Proposed			
		Accuracy ± Std	F1 score ± Std	mAP ± Std	mAR ± Std	Accuracy ± Std	F1 score ± Std	mAP ± Std	mAR ± Std
Conv6-2	50,176	75.86 ± 4.03	78.62 ± 1.28	81.64 ± 0.35	75.85 ± 2.69	87.15 ± 1.02	87.61 ± 0.04	88.85 ± 0.81	86.40 ± 0.85
Conv7-2	50,176	77.13 ± 3.61	79.61 ± 0.73	82.42 ± 0.76	77.02 ± 2.02	88.02 ± 2.78	88.94 ± 1.18	91.20 ± 0.12	86.81 ± 2.36
Conv8-2	25,088	84.39 ± 1.54	84.75 ± 0.69	85.92 ± 0.20	83.62 ± 1.15	89.07 ± 0.10	89.96 ± 0.88	91.24 ± 0.86	88.72 ± 0.91
Conv9-2	25,088	87.10 ± 0.70	87.57 ± 0.47	88.19 ± 0.17	86.97 ± 1.09	89.39 ± 1.10	89.70 ± 1.69	90.24 ± 1.61	89.18 ± 1.76
Avg. pooling	512	89.95 ± 1.26	90.35 ± 1.74	90.72 ± 1.17	89.99 ± 2.29	92.57 ± 0.66	93.41 ± 0.12	94.58 ± 0.37	92.28 ± 0.58

**Table 7:** Performance comparisons of our method (ResNet18 + LSTM) with the conventional ResNet18 with and without PCA (unit: %).

Method	ResNe18 [23]				Proposed			
	Accuracy ± Std	F1 score ± Std	mAP ± Std	mAR ± Std	Accuracy ± Std	F1 score ± Std	mAP ± Std	mAR ± Std
With PCA (No. of eigenvectors = 136)	88.50 ± 1.01	90.16 ± 0.16	91.85 ± 0.11	88.52 ± 0.20	90.01 ± 0.17	91.82 ± 0.37	94.22 ± 0.40	89.54 ± 0.33
Without PCA	89.95 ± 1.26	90.35 ± 1.74	90.72 ± 1.17	89.99 ± 2.29	92.57 ± 0.66	93.41 ± 0.12	94.58 ± 0.37	92.28 ± 0.58

**Table 8:** Comparative classification performance of proposed method and different baseline CNN models (unit: %).

Methods	Accuracy			F1 Score			mAP			mAR		
	Fold 1	Fold 2	Avg. ± Std	Fold 1	Fold 2	Avg. ± Std	Fold 1	Fold 2	Avg. ± Std	Fold 1	Fold 2	Avg. ± Std
SqueezeNet [24]	78.69	77.00	77.84 ± 1.19	77.53	75.95	76.74 ± 1.12	78.38	75.16	76.77 ± 2.27	76.70	76.76	76.73 ± 0.04
AlexNet [16]	79.19	80.97	80.08 ± 1.26	80.31	80.66	80.49 ± 0.24	80.55	80.85	80.70 ± 0.21	80.08	80.47	80.28 ± 0.27
GoogLeNet [8,12,15,17]	83.36	85.82	84.59 ± 1.74	84.99	85.29	85.14 ± 0.21	84.67	85.92	85.29 ± 0.89	85.32	84.66	84.99 ± 0.47
VGG19 [25]	84.81	85.49	85.15 ± 0.48	84.57	86.02	85.29 ± 1.03	85.48	86.27	85.88 ± 0.56	83.67	85.77	84.72 ± 1.48
VGG16 [25]	83.88	87.57	85.72 ± 2.61	84.84	86.77	85.80 ± 1.37	85.20	87.28	86.24 ± 1.47	84.48	86.26	85.37 ± 1.26
InceptionV3 [14,26]	87.23	88.61	87.92 ± 0.98	87.80	89.10	88.45 ± 0.92	86.50	89.24	87.87 ± 1.93	89.14	88.96	89.05 ± 0.13
ResNet50 [23]	88.94	90.17	89.55 ± 0.87	90.13	91.06	90.60 ± 0.66	89.59	91.82	90.70 ± 1.58	90.68	90.32	90.50 ± 0.26
ResNet18 [23]	90.84	89.06	89.95 ± 1.26	91.58	89.13	90.35 ± 1.74	91.55	89.89	90.72 ± 1.17	91.62	88.37	89.99 ± 2.29
Proposed	92.10	93.03	92.57 ± 0.66	93.49	93.33	93.41 ± 0.12	94.32	94.84	94.58 ± 0.37	92.68	91.87	92.28 ± 0.58

**Table 9:** Parametric and structural comparisons of different deep CNN models with our proposed model.

CNN Models	Size (MB)	No. of Conv. Layers	No. of FC Layers	No. of LSTM Layers	Network Depth	Parameters (Millions)	Image Input Size
SqueezeNet [24]	4.6 MB	18			18	1.24	227-by-227
AlexNet [16]	227 MB	5	3		8	61	227-by-227
GoogLeNet [8,12,15,17]	27 MB	21	1		22	7.0	224-by-224
VGG19 [25]	535 MB	16	3		19	144	224-by-224
VGG16 [25]	515 MB	13	3		16	138	224-by-224
InceptionV3 [14,26]	89 MB	47	1		48	23.9	299-by-299
ResNet50 [23]	96 MB	49	1		50	25.6	224-by-224
ResNet18 [23]	44 MB	17	1		18	11.7	224-by-224
**Proposed**	48 MB	17	1	1	19	13.17	224-by-224

**Table 10:** Comparison of classification performance of the proposed method with different handcrafted feature-based methods (unit: %).

Method	Classifiers	Accuracy			F1 Score			mAP			mAR		
		Fold 1	Fold 2	Avg. ± Std	Fold 1	Fold 2	Avg. ± Std	Fold 1	Fold 2	Avg. ± Std	Fold 1	Fold 2	Avg. ± Std
LBP [49]	AdaBoostM2	36.90	34.57	35.74 ± 1.65	28.85	26.55	27.70 ± 1.63	36.90	34.57	35.74 ± 1.65	23.68	21.55	22.61 ± 1.51
	Multi-SVM	45.53	42.15	43.84 ± 2.39	43.34	41.35	42.35 ± 1.41	44.05	41.94	42.99 ± 1.49	42.66	40.77	41.72 ± 1.34
	RF	57.37	56.84	57.10 ± 0.37	53.40	54.31	53.85 ± 0.64	54.53	55.06	54.79 ± 0.37	52.31	53.58	52.95 ± 0.90
	KNN	49.68	51.24	50.46 ± 1.10	46.28	48.44	47.36 ± 1.53	45.73	47.99	46.86 ± 1.59	46.84	48.90	47.87 ± 1.46
HoG [50]	AdaBoostM2	40.28	38.41	39.35 ± 1.33	33.04	32.68	32.86 ± 0.25	40.28	38.41	39.35 ± 1.33	28.00	28.44	28.22 ± 0.31
	Multi-SVM	47.96	51.73	49.84 ± 2.67	51.95	55.66	53.80 ± 2.63	68.13	66.64	67.39 ± 1.05	41.97	47.79	44.88 ± 4.11
	RF	60.10	62.72	61.41 ± 1.85	61.73	64.66	63.19 ± 2.07	68.03	69.29	68.66 ± 0.89	56.49	60.61	58.55 ± 2.91
	KNN	50.14	56.26	53.20 ± 4.33	52.22	57.13	54.68 ± 3.47	57.37	59.45	58.41 ± 1.47	47.93	54.98	51.45 ± 4.99
MLBP [51]	AdaBoostM2	46.42	41.62	44.02 ± 3.40	40.04	34.85	37.45 ± 3.67	46.42	41.62	44.02 ± 3.40	35.20	29.98	32.59 ± 3.69
	Multi-SVM	56.18	54.76	55.47 ± 1.00	53.72	52.49	53.10 ± 0.87	55.70	53.81	54.75 ± 1.33	51.87	51.23	51.55 ± 0.45
	RF	61.56	61.24	61.40 ± 0.22	56.98	58.16	57.57 ± 0.84	58.41	59.75	59.08 ± 0.95	55.62	56.65	56.13 ± 0.73
	KNN	54.38	56.43	55.40 ± 1.45	50.90	53.49	52.20 ± 1.83	50.92	53.21	52.06 ± 1.61	50.88	53.78	52.33 ± 2.05
Proposed		92.10	93.03	92.57 ± 0.66	93.49	93.33	93.41 ± 0.12	94.32	94.84	94.58 ± 0.37	92.68	91.87	92.28 ± 0.58
